# Hard bilateral syphilitic testes with vasculitis: a case report and literature review

**DOI:** 10.1186/s12894-021-00886-5

**Published:** 2021-09-03

**Authors:** Sat Prasad Nepal, Takehiko Nakasato, Takashi Fukagai, Takeshi Shichijo, Jun Morita, Yoshiko Maeda, Kazuhiko Oshinomi, Yoshihiro Nakagami, Tsutomu Unoki, Tetsuo Noguchi, Tatsuki Inoue, Ryosuke Kato, Satoshi Amano, Moyuru Mizunuma, Masahiro Kurokawa, Yoshiki Tsunokawa, Sou Yasuda, Yoshio Ogawa

**Affiliations:** Department of Urology, Showa University School of Medicine, 1-5-8 Hatanodai, Shinagawa-Ku, Tokyo, 142-8555 Japan

**Keywords:** Gumma, Testis, Syphilis, Vasculitis, Aortitis, Hard, Induration

## Abstract

**Background:**

We report the case of a patient with syphilitic testicular gumma and vasculitis with adrenal failure due to chronic steroid use.

**Case presentation:**

A 63-year-old male presented with hard right eye swelling and very firm bilateral testes on palpation, which he had for 2 years. Testicular tumor markers were negative; syphilis test was positive. Radiological examination suggested aortitis and bilateral testicular malignancy. The patient received ampicillin for the infection and prednisolone for vasculitis. Left orchidectomy was performed to confirm the presence of testicular tumor; histological examinations revealed granulomatous orchitis. The prednisolone doses were adjusted because of relapses and adverse effects of steroid use. Unfortunately, the patient died in the intensive care unit because of uncontrolled blood pressure and pneumonia.

**Conclusions:**

This is a rare case of syphilis with testicular involvement and vasculitis. This report shows the importance of broadening the differential diagnoses of testicular firmness.

## Background

Syphilis involvement in the testis is extremely rare, and the literature has very few case reports. Testicular gummata are characterized by multiple swellings in the testis and granulomatous inflammation with a billiard ball-like hard consistency [1]. It is generally associated with patients with human immunodeficiency virus (HIV) infection. A literature search revealed this was the 24th recorded case of testicular syphilis but only the 2nd case of syphilitic vasculitis (aortitis) accompanying testicular gumma. Herein, we discuss a case of testicular gumma with vasculitis and review the current literature regarding the age at presentation, testicular characteristics, and other systemic findings of testicular syphilis.

## Case presentation

In 2014, a 63-year-old patient initially presented at the medicine department with a cough and erythematous areas on the right ankle joint. The patient had a history of smoking (90 pack-years) and chronic obstructive pulmonary disease (COPD).

**Fig. 1:**
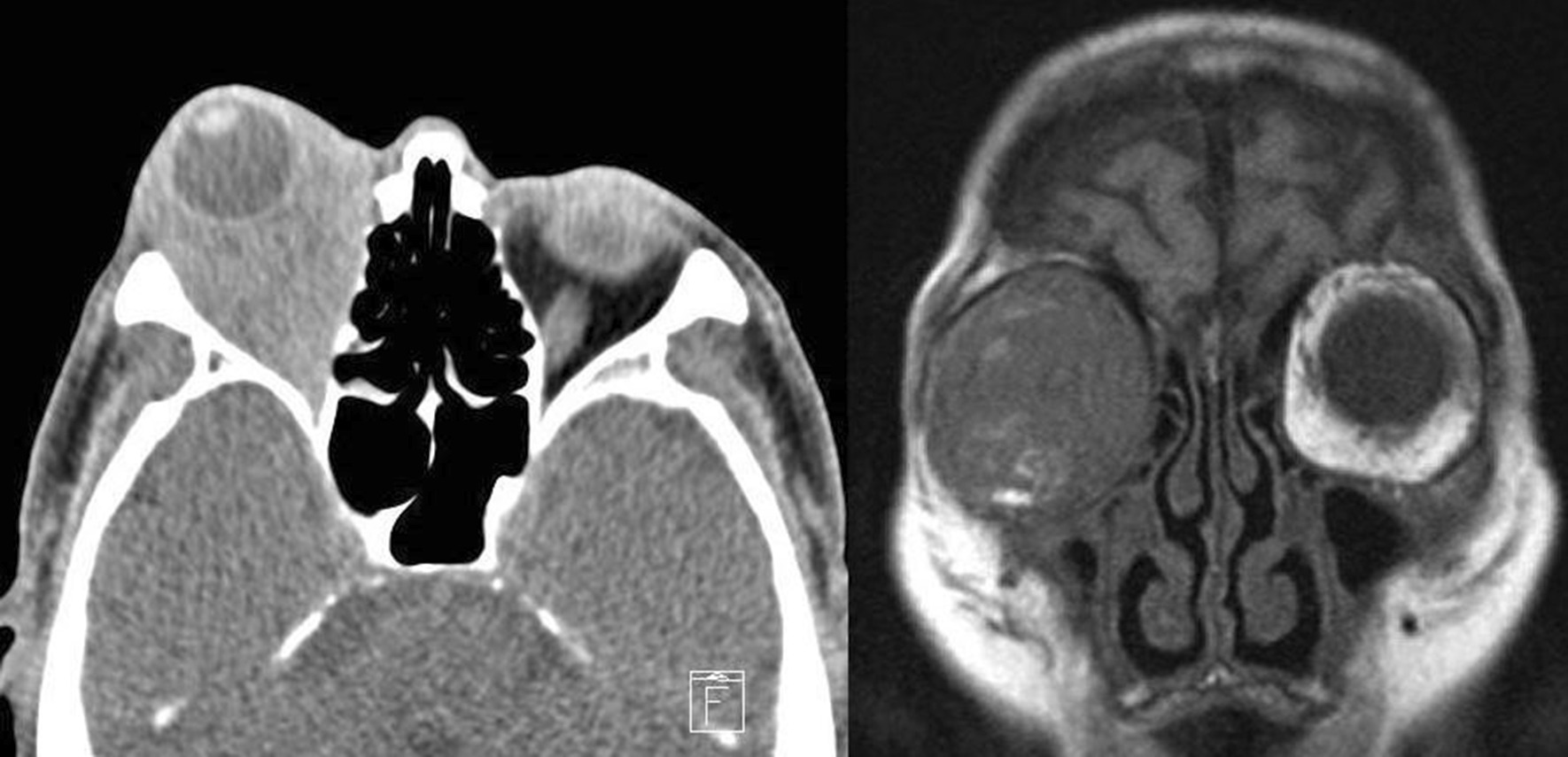
**a** (left) Computed tomography examination of the right orbit showing a soft tissue shadow that occupies the roof of the right eye with no bone abnormality or damage. No abnormalities were noted in the brain parenchyma within the imaging range. **b** (right) Magnetic resonance imaging suggested an intraocular tumor probably because of an inflammatory pseudotumor

On examination, the bilateral testes were painless, very firm, smooth, and nonenlarged. There was bilateral decrease in breathing sounds. A painless right orbital mass—hard and immobile—was also noted, but it did not interfere with normal vision or eye movement. No other abnormal clinical signs were observed (Fig. 1).

Chest X-ray revealed bilateral pleural effusion. Thoracocentesis was normal. Testicular tumor marker levels were not increased. Rapid plasma reagent, *Treponema pallidum* antibody, and absorbed fluorescent treponemal antibody quantitative tests were all positive. Lumbar puncture and magnetic resonance imaging (MRI) were performed, considering the possibility of neurosyphilis. Cerebrospinal fluid findings were normal.

**Fig. 2:**
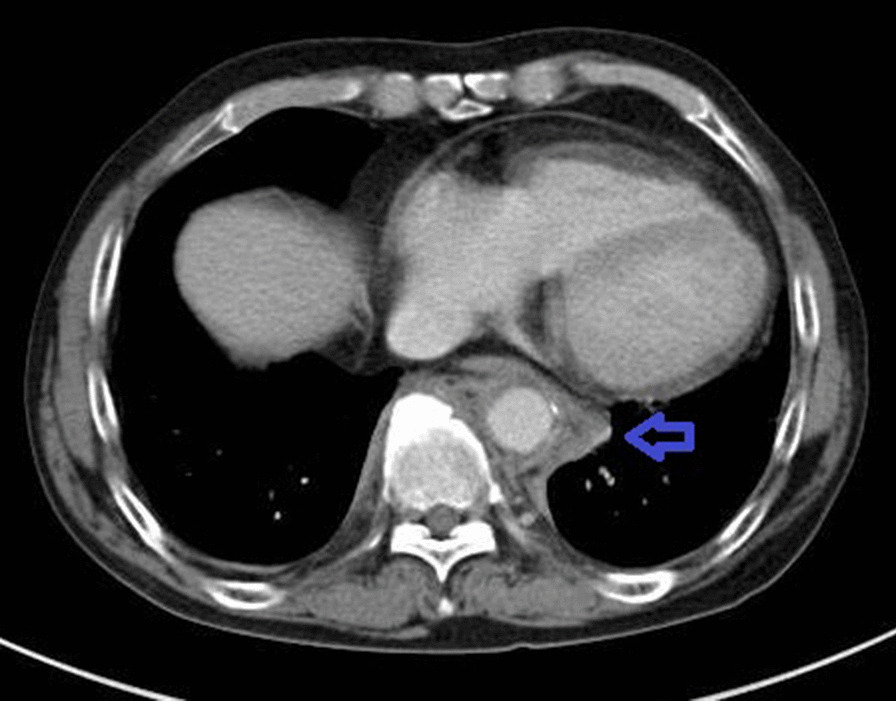
Computed tomography examination of the abdomen showing thickened and edematous areas surrounding the aorta (blue arrow)

**Fig. 3:**
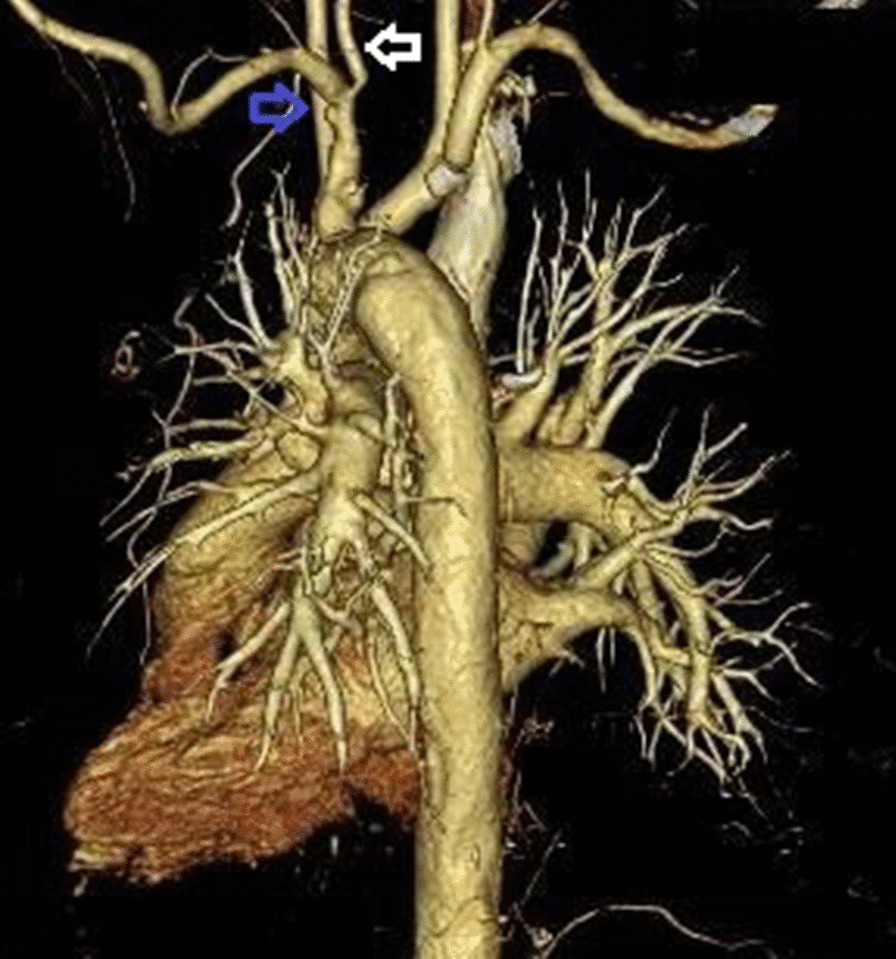
Angiography showing stenosis of the left vertebral artery (white arrow) and left subclavian dilatation (blue arrow)

Computed tomography examination from the aortic arch to the descending aorta revealed an edematous and thickened wall with swollen surroundings. Aortitis was suspected (Fig. 2). Left vertebral artery stenosis and left subclavian artery dilatation were also noted (Fig. 3).

Antineutrophil cytoplasmic antibodies tests were negative. Angiographies of the abdominal cavity, kidney, and superior and inferior mesenteric arteries were performed to assess vasculitis, all of which were negative. An MRI of the ankle was performed because of erythematous lesions in the lower limbs; this revealed inflammatory findings suggesting osteomyelitis. Skin biopsy indicated dermatopanniculitis.

**Fig. 4:**
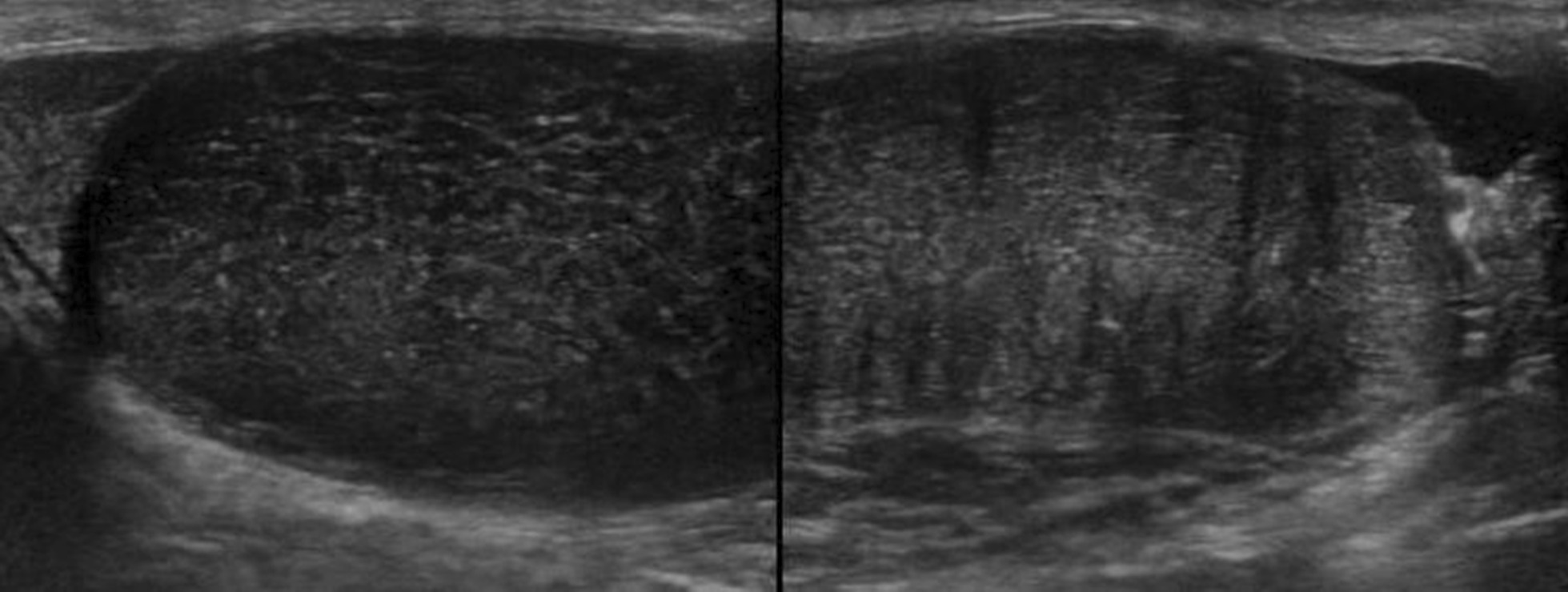
Ultrasonography of bilateral testes showing similar echogenicity

Ultrasound revealed uniform echogenicity across the bilateral testes (right side: 20.3 × 40.8 mm; left side: 26.6 × 42.3 mm). However, the testicular condition was inconclusive (Fig. 4). Therefore, the patient underwent another MRI.

**Fig. 5:**
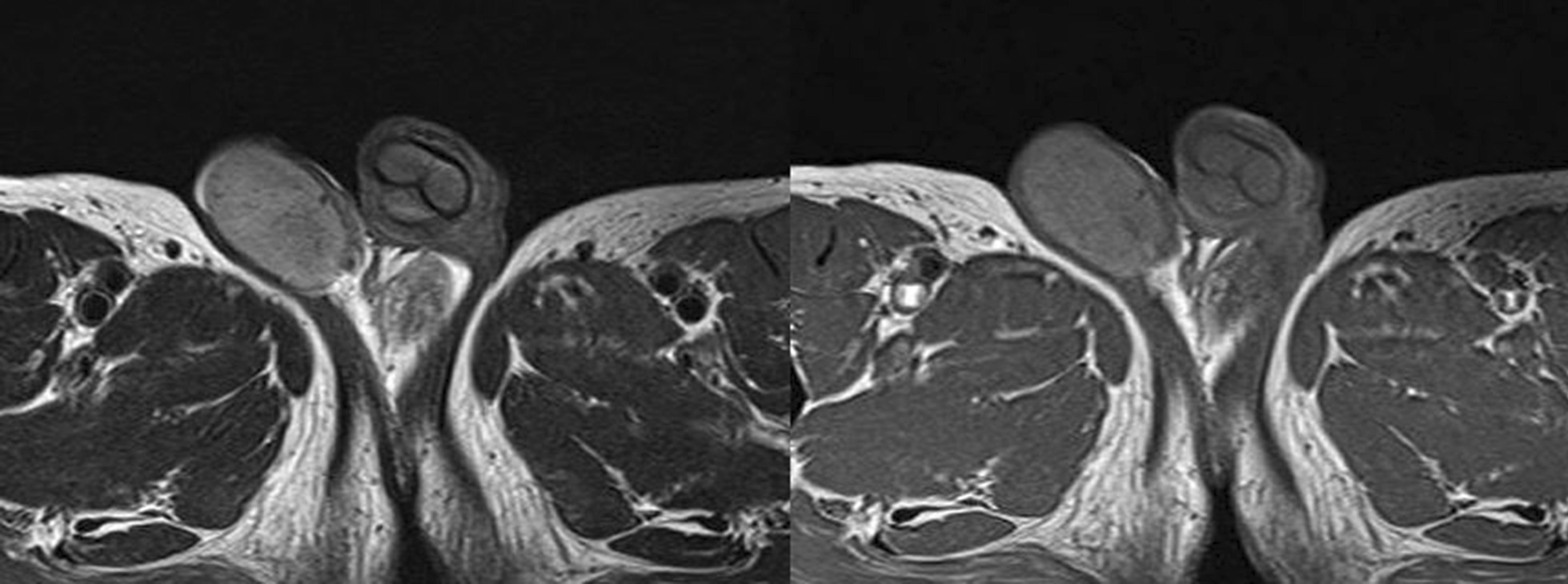
Magnetic resonance imaging of the bilateral testes

Testes MRI showed that the bilateral testes had higher signal than the muscle on T1 imaging and lower signal than that of a normal testis on T2. Thus, malignant lymphoma of the bilateral testes was considered (Fig. 5).

**Fig. 6:**
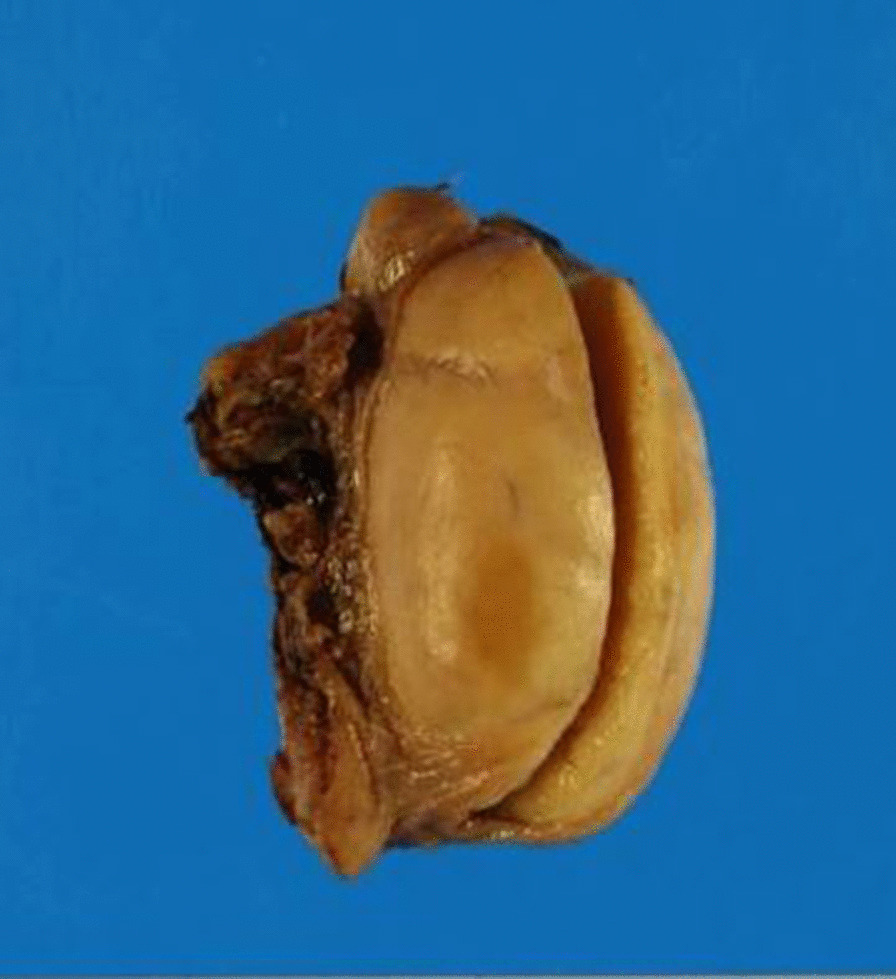
Smooth yellowish left testis

**Fig. 7:**
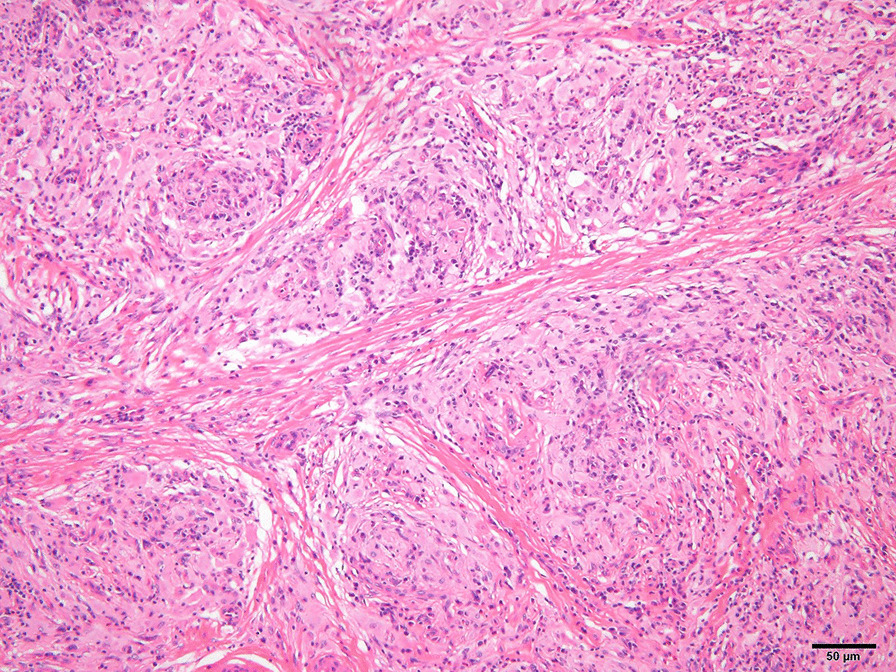
Histology of the testicular gumma, showing epithelioid granulomas. (Horizontal and Vertical resolution at 72 dpi, Magnification × 10)

Left orchidectomy was performed, and the sample was sent for histological examinations. The testis and epididymis had a uniform yellowish-white tumor. The testis was hard in consistency (Fig. 6). Microscopic findings showed that seminiferous tubules were destroyed and surrounded by lymphocytes and plasma cells. CD68-positive epididymal cells were proliferated. No foreign body giant cells or necrotic foci were observed. There were few CD20 (L26)-positive B and CD3-positive T cells; no proliferation of atypical cells was noted. No pathogens were identified on Periodic acid–Schiff, Grocott`s mehtenamine silver and Gram, or Ziehl–Neelsen staining. Granulomatous orchitis was considered (Fig. 7; We used Olympus BX51 Microscope with an objective lens of × 10, Olympus DP73 Camera, and Olympus standard CellSens standard version 1.6 as acquisition software for the microscopy.). Because the histology of left testicle was nonmalignant, the right testicle was not removed.

Our patient was diagnosed with syphilitic gumma with vasculitis. He also had a 30-year-old history of sexual activity with a sex-service worker. Ampicillin (8 g/day) for 2 weeks was started for syphilis treatment. However, prednisolone (0.8 mg/kg/day, 35 mg) was introduced and continued because of a high inflammatory response, vasculitis, and pleural effusion. However, the tests for syphilis were positive even after the antibiotic therapy.

The patient refused treatment for the eye hardness; therefore, no biopsy was performed.

The steroid dose was frequently changed as the patient had relapses (i.e., shortness of breath, pleural effusion, and pericardial fluid accumulation) upon dose reduction and experienced steroid-induced adverse effects (i.e., surgery for avascular necrosis of femoral head, uncontrolled orthostatic hypotension with lower sodium and high potassium possibly due to steroid-induced adrenal insufficiency, and steroid-induced hypogammaglobulinemia).

**Fig. 8:**
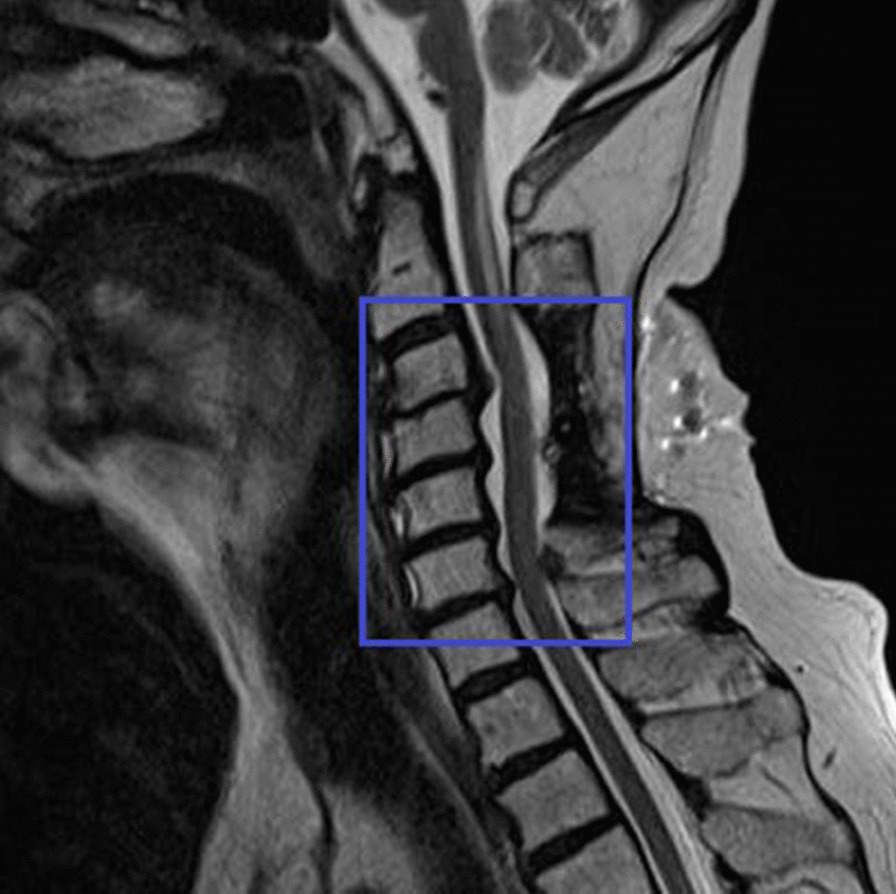
Magnetic resonance imaging of the cervical spine showing C3–C4 cervical spinal canal stenosis due to cervical spondylosis (inside the blue box)

He complained of numbness and tingling sensation in both upper limbs after 2 years of the diagnosis. An MRI of the cervical spine revealed C3–C4 cervical spinal canal stenosis with intervertebral disc swelling and spinal cord compression as well as similar narrowing of the spinal canal in C4–C7 (Fig. 8).

In 2020, the patient was admitted to the intensive care unit (ICU) owing to a deteriorating level of consciousness and uncontrolled blood pressure. He had pneumonia due to cytomegalovirus and *Pseudomonas aeruginosa* infections that led to acute respiratory distress syndrome. Unfortunately, the patient died on day 18 of ICU admission.

## Discussion and conclusions

This report presents a rare case of tertiary syphilis with testicular gumma and syphilitic vasculitis. It could not be controlled with antibiotics and steroids and ultimately led to adrenal dysfunction and mortality. Only a few cases of testicular involvement in syphilis have been reported. Furthermore, testicular induration as the first presentation indicating syphilis is rare.

The indurated right eye may be due to syphilis. However, the reason for the hardened eye mass remains unknown because the patient denied undergoing a biopsy. Ocular syphilis can occur in any stage of syphilis and can infect any part of the eye, with panuveitis being the most common finding. Patients generally present with loss of vision, eye pain, floaters, and photophobia [2]. Orbital involvement is rare and characterized by gumma within the orbit, extraocular muscle, or lacrimal gland and the presence of periostitis [2–4].

The literature review revealed that the mean age of patients with testicular syphilis at disease presentation is 42 years and five patients had HIV infection (Table 1). In total, 14 patients (14/18, 77.7%; we were unable to collect the data of six patients mentioned in the study by Archimbaud et al. [5]) presented with enlarged testis or scrotal swelling. Six patients (6/18, 33.3%) presented with a firm or hard testicle accompanied by testicular swelling. Our patient was unique in presenting with only hard bilateral testes.

**Table 1:** Relevant literature on testicular involvement in syphilis

Case number	Authors	Age (years)	Testicular findings	Systemic findings
1	Lees et al. 1937 [15]	27	Bilateral (first right scrotal swelling, then left scrotal swelling) Congenital syphilis	NA
2	London et al. 1947 [17]	33	Hardening with painful swelling of testes	NA
3	Al-Egaily et al. 1977 [1]	37	Bilateral firm and enlarged testis with painless penile sores	NA
4–9	Archimbaud et al. 1984 [5]		Six cases	NA
10	Onishi et al. 1987 [18]	72	Right scrotal swelling with tenderness	Aortic aneurysm Aortitis
11	Terao et al. 1993 [19]	44	Enlarged firm right testis	NA
12	Nakano et al. 1999 [20]	75	Painless left scrotal swelling	NA
13	Varma et al. 2009 [21]	39	Right testis painful and firm lump	HIV
14	Silva et al. 2010 [22]	32	Bilateral testicular swelling	NA
15	Nakano et al. 2011 [8]	47	Painful right testicular swelling	NA
16	Sekita et al. 2012 [23]	40	Left scrotal swelling Left testis	NA
17	Teo et al. 2012 [14]	47	Right testis with firm, non-tender swelling Non-ulcerated indurated subprepuce Conservative treatment with doxycycline	NA
18	Liang et al. 2013 [24]	37	Left testicle and left kidney Painless swelling Doxycycline coz of penicillin allergy post-operation	HIV
19	Yogo et al. 2014 [25]	28	Right testis pain and swelling Jarisch–Herxheimer reaction in the testis following Penicillin G infusion	HIV with Bilateral Retinal detachment
20	Morlacco et al. 2015 [26]	31	Right testis pain and swelling	NA
21	Chu et al. 2016 [27]	33	Right testis hardening and swelling Non-granulomatous type	HIV
22	Tagliati et al. 2020 [13]	39	Testicular discomfort Multiple bilateral subcentimetric hypoechoic lesions	NA
23	Agrawal et al. 2020 [28]	40	Left scrotal abscess due to epididymo-orchitis Ulcerated enlarged left testis with indurated base	HIV
24	Our case	63	Bilateral testes induration	Aortitis Left subclavian artery aneurysm

*HIV* human immunodeficiency virus, *NA* not applicable

The incidence of syphilis is currently increasing in developed countries. More than half of patients are males are owing to men having sex with men, and 42% of such patients were also HIV-positive [6]. The number of patients with syphilis is increasing by 1100 per year in Japan [7]; however, it is mainly because of heterosexual transmission rather than homosexual transmission [8]. It can also be caused by a direct *T. pallidum* (subspecies *pallidum*) infection and then is transmitted via blood products, either transplacentally or sexually.

Syphilis has three stages: primary (characterized by painless chancre occurring 2–6 weeks after infection), secondary (characterized by condylomata lata 1–2 months after primary syphilis), and tertiary (occurring 2–50 years after the initial infection, characterized by gummatous disease, meningovascular disease, tabes dorsalis, cardiovascular, ocular, and otic syphilis) [6].

Serologic testing is currently the standard approach for diagnosis; however, it lacks sensitivity in detecting early syphilis, congenital syphilis, neurosyphilis, tertiary syphilis, and HIV or Hepatitis C coinfection [9]. Hence, several other approaches such as immunohistochemistry (IHC), polymerase chain reaction (PCR), culture, morphological observation, and seroassay are considered for the detection of clinically undetected syphilis [10]. In a previous study, IHC had 49–92% sensitivity and excellent specificity for the diagnosis of secondary syphilis [9]. It can be used as a tool for further investigation when serological assays fail to identify the organism. However, there is a possibility of cross reaction with *Borrelia burgdorferi* and intestinal spirochetes [10]. PCR has a sensitivity of 89.1% in chancre specimens from patients with primary syphilis [10, 11]. In fact, the United States Centers for Disease Control and Prevention suggests that PCR is valuable for chancre samples [11]

Syphilitic gummata can mimic testicular tumors, which are usually diagnosed after surgery. Its differential diagnoses such as testicular neoplasms, mumps orchitis, tubercular epididymitis, and gonococcal epididymitis should be considered [1]. Gummata are usually multiple and regressive. It is clinically diagnosed with syphilis serology and treatment response [1]. On biopsy, obliterative endarteritis with palisading lymphocytes and plasma cells is visible. Over time, fibrous scarring causes tubular atrophy and sterility [1].

Ultrasonography shows lesions to be cystic with increased peripheral vascularity [12]. On contrast-enhanced ultrasonography, lesions show rapid wash-in and early washout [13]. Moreover, orchidectomy is generally performed for testicular syphilitic masses because of the concerns regarding missing out on the testicular tumor, thus requiring conservative management [13–15].

Penicillin G is the antibiotic of choice for syphilis. However, ampicillin treatment results in a higher cerebrospinal fluid concentration in the brain than penicillin G treatment. It was used in our patient to avoid neurosyphilis, although the investigations for this were negative [8]. Although intramuscular benzathine penicillin G is the first-line antibiotic recommended by the World Health Organization, it is unavailable in Japan and carries a risk of anaphylaxis [16]. Unfortunately, the syphilis tests were not negative in our patient even after the antibiotic therapy. Thus, therapy was inadequate due to severe inflammation.

The present case highlights the importance of broadening the differential diagnoses of testicular hardening or indurations. Patients with syphilitic gummata present only with hardness; this needs to be considered for timely treatment of and complication prevention in patients with similar presentations.

## Data Availability

Records and data concerning the case are stored in Showa University Hospital medical records. To obtain access to the raw data, please apply for permission to the Department of Urology, Showa University.

## Abbreviations

COPD: Chronic obstructive pulmonary disease
HIV: Human immunodeficiency virus
ICU: Intensive care unit
MRI: Magnetic resonance imaging
